# Lysine Acetyltransferase 6 in Health and Disease

**DOI:** 10.1002/mco2.70520

**Published:** 2025-12-04

**Authors:** Yujing Tan, Jiani Wang, Fei Ma

**Affiliations:** ^1^ Department of Medical Oncology National Cancer Center/National Clinical Research Center for Cancer/Cancer Hospital Chinese Academy of Medical Sciences & Peking Union Medical College Beijing China

**Keywords:** breast cancer, KAT, KAT6A, KAT6B, KAT6 inhibitor

## Abstract

Lysine acetyltransferase 6 (KAT6) consists of KAT6A and its paralog KAT6B, which represent crucial regulators for epigenetic modifications. By acylating histone H3 and nonhistone proteins, KAT6 enzymes play predominant roles in transcription, cell cycle, diverse developmental processes, regulation of the immune system, and self‐renewal and maintenance of hematopoietic and neural stem cells. Importantly, the frequent molecular dysregulation of KAT6A and KAT6B correlates with survival outcomes of cancers, contributing to the exploration of a wide array of small‐molecule inhibitors against KAT6 catalytic activity. Recent progress in drug discovery has led to the development of dual KAT6A and KAT6B inhibitors with potent antitumor efficacy and selectivity in both preclinical and clinical settings, supporting KAT6 as a druggable, promising target for the treatment of cancers, particularly breast cancers. In this review, we summarize the currently available information regarding the physiological and pathological functions of KAT6A and KAT6B and discuss their potential as antitumor targets in drug development. We also present the discovery and development of an emerging class of KAT6 inhibitors under investigation for breast cancer, along with potential molecular mechanisms underlying the therapeutic efficacy of targeting KAT6, providing references for developing therapeutic strategies in clinical practice.

## Graphical abstract

KAT6A and its paralog KAT6B have emerged as druggable targets for the treatment of malignancies, especially for breast cancer. Recent progress in drug discovery has promoted the development of dual inhibitors targeting KAT6A and KTA6B, which shows potent antitumor efficacy and manageable toxicity. In this review, we summarize the currently available information regarding the molecular mechanisms of KAT6 activity, as well as its physiological and pathological roles. Moreover, we present KAT6 modifiers being considered in malignancies, as well as associated biomarkers for the efficacy of KAT6 inhibitors, which could be used to identify potential candidates suitable for KAT6 inhibitors. The review may deepen our understanding of the effects mediated by KAT6 in health and disease, expanding the potential therapeutic strategies that combine KAT6 inhibitors for the treatment of malignant diseases.

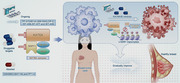

## 1 Introduction

Epigenetic modifications are defined as heritable alterations in gene expression or cellular phenotype that do not entail changes in the DNA sequence [1, 2, 3, 4, 5]. These modifications regulate gene transcription and fundamental cellular processes at several levels, including DNA methylation, chromatin remodeling, expression of noncoding RNAs, and posttranslational modifications of histones [6, 7, 8, 9]. Some epigenetic changes are relatively conserved across generations and are enzyme mediated, with the potential for reversal through specific epigenetic modifiers [10, 11]. Research indicates that dysregulation of epigenetic regulatory functions represents a significant cause of tumor plasticity associated with cancer cell survival, metastasis and drug resistance [12, 13, 14]. Consequently, targeting epigenetic modifications offers a promising avenue for precision therapy of cancers.

**FIGURE 1:**
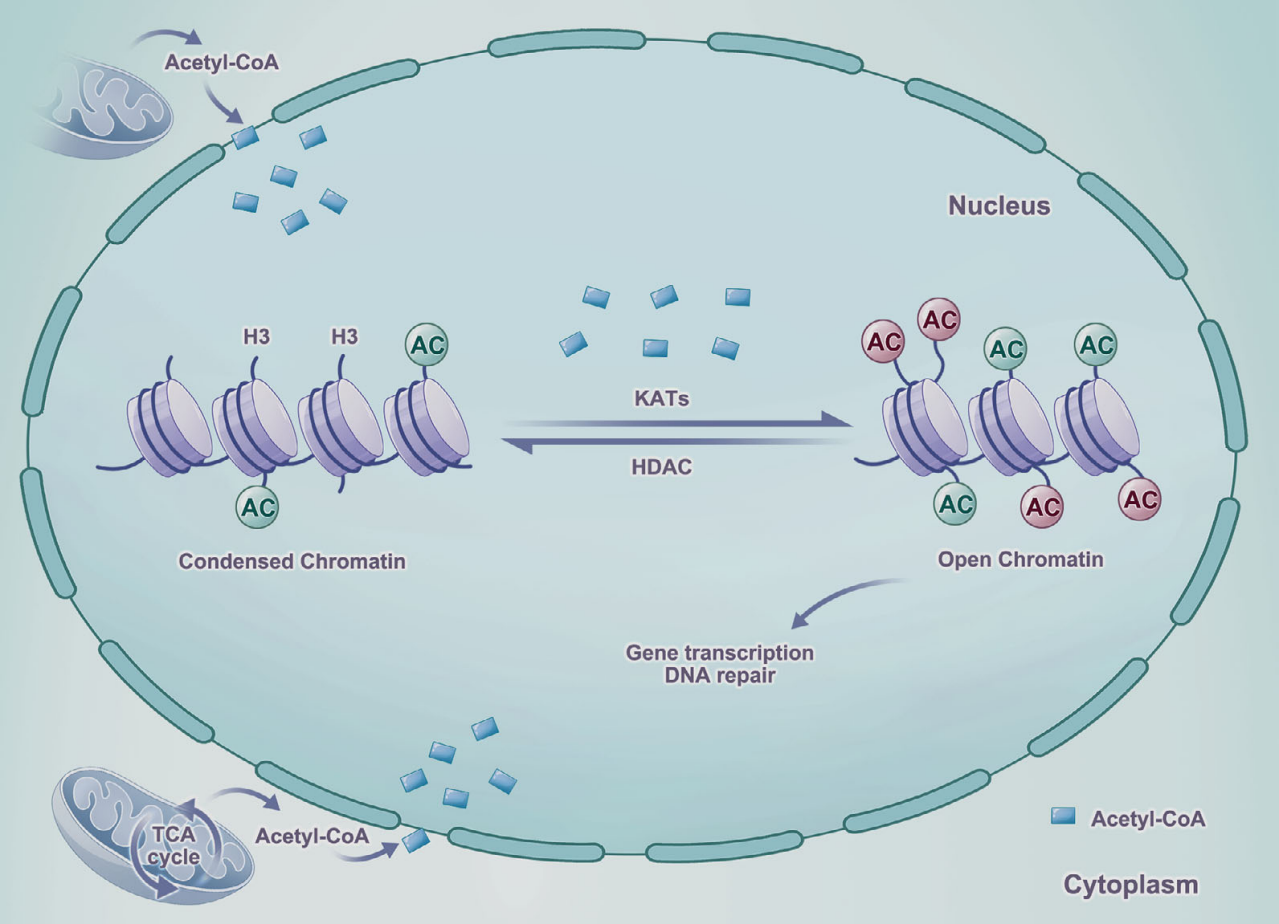
The schematic diagram of acetylation and deacetylation. In the nucleus, histone acetyltransferases (HATs) catalyze acetylation, while histone deacetylases (HDACs) catalyze deacetylation. HATs physically relax chromatin and activate DNA transcription through the acetyl donor of acetyl‐coenzyme A (acetyl‐CoA). In contrast, HDACs restore a compact and ordered nucleosome structure, leading to a closed state for transcription.

Posttranslational histone modifications, including methylation, acetylation, phosphorylation, SUMOylation, ADP‐ribosylation, and ubiquitination, regulate gene expression activation by modulating chromatin accessibility for DNA transcription [15, 16, 17]. Three types of functional proteins are involved in the process, including “writers” (perform histone modifications and serve as epigenetic markers), “readers” (read histone modifications), and “erasers” (erase histone modifications). Histone acetylation and deacetylation represent the most pervasive regulatory mechanisms of histone modifications [16, 17, 18]. This dynamic and reversible process is catalyzed by specific enzymes. Acetylation is catalyzed by histone acetyltransferases (HATs) that are commonly referred to as lysine acetyltransferases (KATs), while deacetylation is catalyzed by histone deacetylases (HDACs) [17, 19, 20]. HATs promote the relaxation of chromatin and provide a plethora of DNA‐binding sites for transcription, through the acetyl donor of acetyl‐coenzyme A (acetyl‐CoA) [21, 22]. The activation of HDACs can reverse this process by removing the attached acetyl groups, which is facilitated by the recruitment of corepressor factors and the restoration of a compact and ordered nucleosome structure that prevents the access of transcription factors [19, 21, 23] (Figure 1).

According to intracellular localization, HATs can be categorized as A‐type and B‐type [24]. A‐type HAT is located in the cell nucleus, where it acetylates histones and a series of chromatin‐related proteins, thereby impacting gene transcription patterns and functions [23, 24, 25, 26]. Conversely, B‐type HAT catalyzes the acetylation of newly synthesized histones in the cytoplasm, which places no direct impact on transcription [24]. KATs catalyze acetylation following the fundamental routine as observed in A‐type HATs [15, 23], indicating their role in transcriptional activity. Frequent molecular dysregulation within KAT enzymes results in alterations of active enzymatic forms at stoichiometric levels and subsequent impact on histone acetylation at global levels, which is responsible for gene‐specific alterations in transcription of oncogenic and tumor suppressor genes [15, 17, 27]. The most common site for such molecular alterations in KAT enzymes is found in paralogous enzymatic subunits within KAT complexes, including KAT6A and KAT6B [28].

Molecular dysregulation of KAT6A and KAT6B has been observed in various tumor diseases, including solid tumors of the breast, lung, and ovary, as well as hematological tumors such as acute myeloid leukemia (AML) [29, 30, 31, 32, 33, 34, 35]. This dysregulation has been reported to correlate with survival outcomes in several cancers [34, 35]. For instance, KAT6A upregulation was identified in estrogen receptor‐positive (ER+) breast cancer, which was associated with progressive development and poor clinical outcomes [32, 34]. Moreover, preclinical data from cell lines and animal models indicate that approaches targeting subunits of KAT6A and KAT6B can effectively inhibit tumor growth via mechanisms including cell cycle arrest and cell senescence [36]. Recently, a phase I clinical trial demonstrated that PF‐07248144, a dual KAT6A and KAT6B inhibitor, in combination with endocrine therapy (ET), exhibited promising antitumor efficacy and manageable safety profiles in patients with ER+/human epidermal growth factor receptor 2‐negative (HER2−) (ER+/HER2−) advanced breast cancer (ABC) following standard treatment with cyclin‐dependent kinases 4/6 inhibitor (CDK4/6i) [37]. These findings provide a theoretical proof of concept that KAT6A and KAT6B are promising druggable targets for epigenetic drugs in the treatment of ER+ breast cancer.

In the current review, we summarize the existing knowledge regarding molecular mechanisms of KAT6 activity and crucial roles of KAT6 in physiological and pathological processes. More importantly, we discuss the potential of therapeutic targeting of KAT6 and present KAT6 modifiers being considered in malignancies, as well as associated biomarkers for the efficacy of KAT6 inhibitors, which could be employed to identify candidates suitable for KAT6 inhibitors. By doing so, we seek to deepen our comprehension of effects mediated by KAT6 in health and disease and to explore the potential therapeutic strategies of KAT6 inhibitors for the treatment of malignant diseases.

## 2 Molecular Mechanisms of KAT6 Activity

### 2.1 Enzymatic Function and Substrate Specificity

The paralogous KAT6A (formerly known as MOZ and MYST3) and KAT6B (formerly known as MORF and MYST4) were first discovered approximately 30 years ago. They belong to the MYST family, which encompasses other members of KAT5 (also designated TIP60), KAT7 (also designated HBO1), and KAT8 (also designated MOF) [38, 39, 40].

**FIGURE 2:**
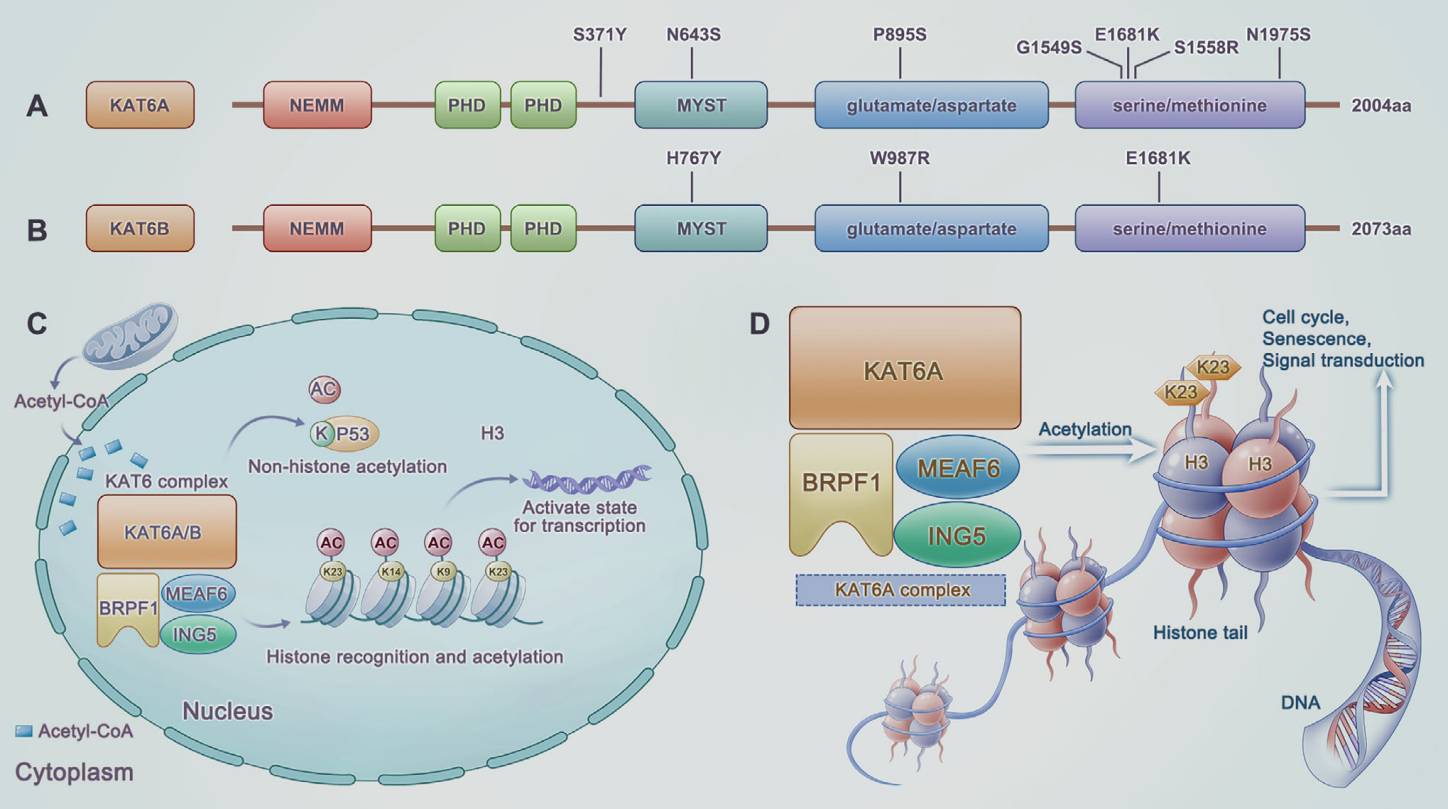
The schematic diagram of KAT6. Domain organization of KAT6A (A) and KAT6B (B), shares 60% amino acid identity and 66% similarity. It comprises an N‐terminal part of the NEMM domain, a MYST domain, a double PHD zinc finger domain, and a C‐terminal part of a glutamate/aspartate‐ and a serine/methionine‐rich region. Pathogenic missense mutations in KAT6A and KAT6B were presented. (C) KAT6A protein functions through the formation of a tetrameric complex, which is comprised of KAT6A, BRPF1, ING5, and MEAF6. It utilizes acetyl‐CoA as the acetyl donor and facilitates the acetylation of histones (H3K9, H3K14, and H3K23) and nonhistone proteins (p53), leading to the physical relaxation of chromatin and subsequent activation of DNA transcription. (D) Through the regulation of chromatin structure, the KAT6 complex participates in a wide range of biological processes, including cell cycle, cell senescence, and so on.

The KAT6A gene is located in chromosome 8p11‐12, and the KAT6B gene is located in chromosome 10q22‐2. KAT6A and KAT6B proteins are composed of 2004 and 2073 amino acids, respectively, of which they share 60% amino acid identity and 66% similarity [29]. In terms of domain organization, it consists of an N‐terminal part of Enok, MOZ, or MORF (also called NEMM domain), a MYST domain, a double plant homeodomain (PHD) zinc finger domain, and a lengthy unstructured C‐terminal part that can be further subdivided into a glutamate/aspartate‐ and a serine/methionine‐rich region. The NEMM domain (for KAT6A), as well as the PHD zinc finger domain (for KAT6A and KAT6B), is conserved, which was shown to play a crucial role in nuclear localization [41, 42, 43]. The MYST domain of KAT6A is a catalytic domain that can bind to DNA through the PHD zinc finger (bind to histone H3) and helix‐turn‐helix motifs, thus promoting acetylation [44]. The C‐terminal region was revealed to regulate transcriptional activity for activation [29, 45] (Figure 2A,B).

### 2.2 KAT6 Complexes and Interacting Partners

Frequently, KAT6A or KAT6B protein exerts biological functions in the context of the formation of a tetrameric acetyltransferase complex with other chromatin‐related proteins, but not appearing in isolation. These chromatin‐related proteins comprise different bromodomain (BRD)‐PHD finger proteins (BRPF1/2/3), ING5, and MEAF6 (also known as EAF6) [39, 42, 46] (Figure 2C,D). Among the three types of BRPF proteins (BRPF1/2/3), BRPF1 occupies a dominant position in the assembly and activation of the KAT6A complex, as it can preferentially enrich in KAT6A protein [47, 48, 49]. Moreover, the BRPF1 subunit, as well as the ING5 subunit, serves as readers of histone modification, which probably improves the apparent affinity and specificity of the KAT6 complex [47, 48]. Through interaction among the other three subunits, KAT6A or KAT6B forms the tetrameric complex to acetylate lysine residues on histone H3 tails, which results in a physically relaxed chromatin that is suitable for DNA transcription.

In addition to acetylation, KAT6A and KAT6B complexes have been demonstrated to play an important role in several recently appreciated types of histone modifications, including crotonylation, butyrylation, and propionylation. They can read these histone acylations through the double PHD finger domain, as well as write histone propionylation by the MYST domain [50, 51, 52, 53]. For example, KAT6 complexes were reported to be essential for propionylation of histone lysine residues in vitro and in vivo in mouse embryos [50]. In another mouse model for KAT6A–TIF2‐driven leukemia, high transcription levels of KAT6A were shown to correlate with enrichment of histone H3 propionylation at lysine 23 [51].

### 2.3 Regulation of Gene Expression Programs

By utilizing acetyl‐CoA as the acetyl donor, the KAT6 complex modulates chromatin organization, which is achieved by the ε‐amino acetylation of lysine residues at the N‐terminal tails of core histones. Particularly, KAT6 acetyltransferase complexes recognize and catalyze acetylation of lysine 9, 14, and 23 on histone H3 (H3K9, H3K14, H3K23), which is correlated with an active state for DNA transcription [23, 54, 55, 56]. Among them, H3K23 is the primary regulator for acetylation mediated by KAT6A.

Apart from histones, KAT6 complexes acetylate a diverse range of nonhistone proteins, including p53 and SMAD3 [57, 58, 59]. In experimental models of tongue squamous cell carcinoma (TSCC), KAT6B has been found to act as a direct functional target of miR‐22, whose activation relies on the intensity of stresses in the presence of p53, suggesting the role of KAT6B in the activation of p53 [59]. The association between SMAD3 and KAT6A has also been demonstrated in triple‐negative breast cancer (TNBC). KAT6A involves the acetylation of SMAD3 at K20 and K117, which promotes SMAD3 interaction with an oncogenic nuclear receptor binding protein TRIM24, and attenuates SMAD3 interaction with the tumor suppressor TRIM33. As expected, the process potentiates the cell proliferation and metastasis of TNBC via a potential mechanism of increased cancer stem‐like cell stemness and myeloid‐derived suppressor cell recruitment [57].

By modulating the acetylation of histones and nonhistone proteins, the KAT6 complex is involved in a multitude of cellular events through the regulation of chromatin accessibility for DNA transcription. These events include transcriptional control, chromatin organization, cell cycle, cell senescence, cell differentiation, and signal transduction [60, 61, 62, 63, 64, 65] (Figure 2C,D).

## 3 KAT6 in Physiological Processes

As one of the crucial enzymes in epigenetic modifications, the KAT6 enzyme is involved in multiple biological processes, including embryonic development [66, 67, 68], maintenance of neural stem cells (NSCs) and hematopoietic stem cells (HSCs) [56, 69, 70, 71, 72], as well as regulation of the immune system [73, 74, 75, 76, 77].

### 3.1 Embryonic Development

Considering the crucial role of KAT6 acetyltransferases in a large array of histone and nonhistone modifications, which is associated with diverse gene transcription patterns and functions, it is reasonable that KAT6A and KAT6B will have an impact on embryonic survival. Indeed, studies have shown their roles in multiple processes associated with embryonic development [68, 70, 78].

Mice homozygous for a KAT6A deletion exhibited embryonic lethality, indicating its critical role in embryonic development. It was accompanied by a series of developmental defects, including craniofacial abnormalities, impaired regulation of HSCs, heart failure, deficient development of body segment identity, and skeletogenesis [34, 60, 66]. The mechanism may be related to the change in HOX gene expression. HOXA9 expression has been detected to be reduced in KAT6A‐deficient fetal hepatocytes, which is featured by a decreased number of HSCs and B lymphocytes [78]. Through insertion of the neomycin phosphotransferase gene into exon 16, leading to death of homozygous KAT6A‐mutant mice [70]. Furthermore, enhanced methylation and transcriptional repression at the HOX locus have been observed in these mice, which is correlated with H3K9 hypoacetylation [70]. It resulted in cervical extension, additional cervical vertebrae, and fewer thoracic vertebrae [68].

Similarly, KAT6B deficiency or KAT6B harboring loss of function (LoF) mutations resulted in defects in the development of central nervous system, and failure to thrive in the postnatal period [66]. Concurrently, KAT6A and KAT6B play a significant role in the development of the cardiac structure, particularly in the ventricular septum [67]. Mice with homozygous deficiency of the KAT6A or KAT6B gene exhibited a high prevalence of ventricular septal defects and other congenital cardiac anomalies. Up to 50% of mice with KAT6A or KAT6B gene knockout exhibit cardiac defects [79, 80].

To summarize, KAT6 has a pivotal effect on embryonic development under normal physiological conditions, while the deficiency of KAT6A and KAT6B generally contributes to embryonic lethality due to various forms of developmental defects.

### 3.2 Adult Tissue Homeostasis

KAT6A and KAT6B play a crucial role in maintaining adult tissue homeostasis, which is achieved through two key aspects: regulating stem cells and the immune system.

#### 3.2.1 Maintenance and Renewal of HSCs and NSCs

The structural and biochemical similarities between KAT6A and KAT6B result in comparable biological effects on stem cell development. Nevertheless, there appear to be several discrepancies, with KAT6A being referenced more frequently in the context of maintaining and renewing HSCs, whereas KAT6B has been the subject of greater investigation in the field of neural development.

KAT6A exerts a profound influence on the process of hematopoiesis, largely due to its indispensability in the self‐renewal and proliferation of HSCs. The activity of KAT6A was shown to be required for the renewal of HSCs and NSCs, as it can effectively prevent stem cells from entering senescence by the INK4A–ARF pathway [71, 81]. Additionally, KAT6A was found to favor the generation and maintenance of HSCs and the appropriate development of the hematopoietic system, including erythroid, myeloid, and B‐lineage cell progenitors [82]. This process was associated with the activation of a series of sequence‐specific transcription factors related to hematopoiesis, such as AML1 (RUNX1) and PU.1 [78, 83, 84]. Theoretically, KAT6A inhibition may potentially lead to impaired hematopoietic function. This may explain why the sole in‐human clinical trial associated with the KAT6 inhibitor PF‐07248144 revealed a high prevalence of neutropenia [37]. In addition, KAT6 inhibitors currently under investigation demonstrate a tendency toward combined application with CDK4/6i, such as PF‐07248144, ISM5043, and OP‐2. It has come to realization that neutropenia constitutes the most common adverse event (AE) during the treatment with palbociclib and ribociclib [85, 86, 87]. The concomitant administration of analogous drugs with similar AEs may result in an increased frequency or severity of bone marrow suppression. In prospective clinical trials, it is recommended that close monitoring of patients’ blood count be conducted. This allows for the timely adjustment of drug dosage or treatment strategies to minimize or prevent the occurrence of severe AEs.

KAT6B plays a regulatory role in NSCs and the process of neurogenesis. KAT6B deficiency exhibited a reduced efficiency in differentiating to neural lineage, involving a mechanism associated with chromatin plasticity and differentiation toward neural progenitor cells during neural development [66, 72, 88, 89, 90]. In detail, KAT6B knockout physically compacts the chromatin organization, leading to reduced expression of SOX1 as well as weakened interactions of pluripotency transcription factors Oct4 and Nanog with chromatin [72]. As a neural progenitor biomarker, SOX1 maintains neural cells in an undifferentiated state and regulates pluripotency, self‐renewal, and differentiation [91, 92]. Oct4 and Nanog are crucial mediators of self‐renewal and the maintenance of pluripotency in undifferentiated embryonic stem cells [72].

Therefore, KAT6 has a significant impact on the maintenance and renewal of HSCs and NSCs, with KAT6A being more critical in regulating HSCs, while KAT6B has a higher importance in regulating NSCs.

#### 3.2.2 Immune Regulation

KAT6A and KAT6B play a notable role in the immune system, with effects observed in the activation of macrophages, the production of inflammation‐associated cytokines, and the proliferation of B cells and T cells.

The differentiation of monocytes and ensuing polarization of macrophages are essential for inflammation, which is vital for the defense against pathogens and the maintenance of physiological equilibrium. Macrophages can be polarized into two functionally different phenotypes of M1 and M2 macrophages, where M1 macrophages are a type of classically activated macrophage and display proinflammatory effects, whereas M2 macrophages weaken the inflammatory activity and promote tissue repair [93, 94]. The dysregulation of KAT6A and KAT6B has been demonstrated to modulate the process of macrophage polarization. In mice models, KAT6A overexpression facilitated the differentiation of monocytes into macrophages, the polarization of M1 macrophages, and the optimal phagocytosis activity of M1 macrophages. Concomitantly, the levels of proinflammatory cytokines, including IL‐1β, tumor necrosis factor (TNF)‐α, and IL‐6, were found to increase in conjunction with M1 macrophage polarization when KAT6A was highly expressed. KAT6A inhibition reduced histone acetylation in the enhancers of genes encoding the aforementioned proinflammatory cytokines (including IL1B and TNF‐α), as well as M1 macrophage polarization [73, 95]. In contrast with the function of KAT6A in activating macrophages, KAT6B was shown to be strongly downregulated in classically activated M1 macrophages [73]. To say, KAT6A upregulation promotes M1 macrophage polarization, which results in a series of proinflammatory reactions, while KAT6B tends to polarize macrophages toward the M2‐type macrophage which inhibits inflammation. This suggests that the roles of KAT6A and KAT6B in macrophage polarization operate in opposite directions.

In addition to modulating macrophage activity, KAT6A has been found to shape functional diversity and modulate immune responses of T‐cell subsets. Specifically, homozygous deficiency of KAT6A decreased surface CD8 coreceptor expression through the impact of H3K9Ac, which resulted in a robust reduction of T cell receptor signaling intensity and impairment of the effector‐like memory compartment. The removal of the E8I enhancer from the CD8 coreceptor gene can reverse this process [74]. Moreover, KAT6A has been shown to have an impact on the functional outcome of humoral immune responses in B cells. KAT6A defects promoted the differentiation of germinal center B cells into IgM and low‐affinity IgG1(+) memory B cells, reducing secondary high‐affinity antibody‐secreting cell formation [75].

Recently, KAT6A has been identified as a crucial factor in the proliferation and differentiation of proinflammatory CD4⁺ T cells, exerting its influence through the epigenetic programming of immunometabolism [76]. Specifically, KAT6A catalyzed histone acetylation at the chromatin and modulated transcriptional activity of several glycolytic genes, thereby orchestrating metabolic reprogramming of glucose in CD4+ T cells and subsequent CD4+ T cell responses [76]. This suggests that KAT6A could serve as a promising target for enhancing immune checkpoint inhibitor (ICI) therapy. HR+ breast cancer cells were classified as the “cold” or “moderate” tumor microenvironment (TME) subtypes, which possessed low immune‐cell infiltration or the presence of stromal cell infiltration [96, 97]. The feature presents a challenge to the development of immunotherapy for HR+ breast cancer, particularly in comparison with “hot” tumors such as TNBC and other solid tumors [96, 97, 98, 99]. The regulatory role of KAT6A in the TME offers insights into the development of novel therapeutic strategies regarding ICI in HR+ breast cancer. Potentially, the addition of KAT6 inhibitors to ICI in HR+ breast cancer may result in the generation of a “hot” TME, characterized by a high percentage of adaptive and active innate immune cells. Further evaluation is required.

## 4 KAT6 in Disease Pathogenesis

**FIGURE 3:**
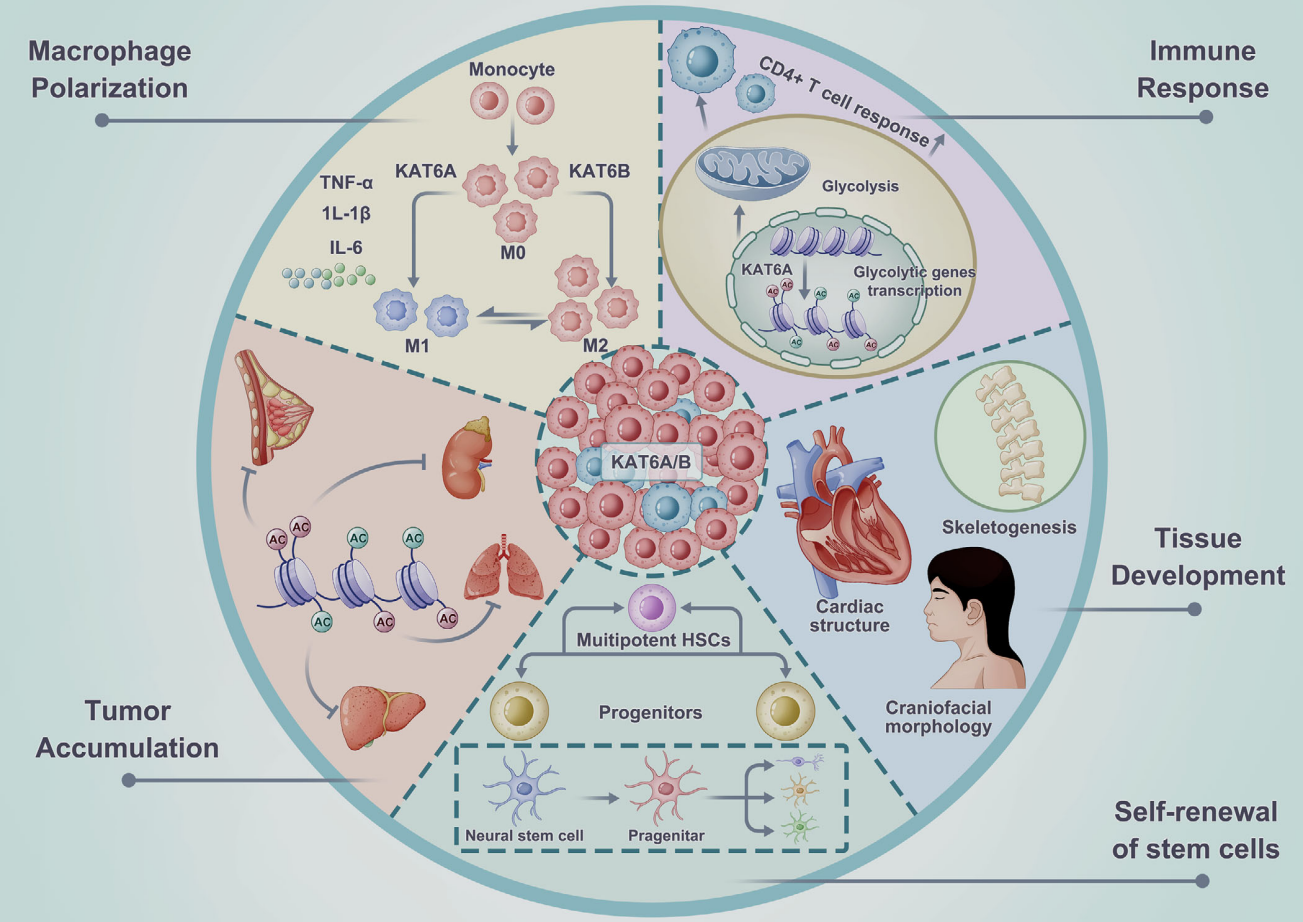
Function of KAT6 in physiology and pathology. As important epigenetic modifiers, KAT6A and KAT6B are involved in multiple processes, including tissue development, self‐renewal, and maintenance of neural stem cells (NSCs) and hematopoietic stem cells (HSCs), regulation of immune response, as well as the onset and development of tumors.

Importantly, abnormal regulation of KAT6A and KAT6B has been shown to serve as tumor drivers or suppressors, which places a critical impact on the formation, progression, and drug resistance of cancerous disorders [30, 31, 32, 100]. Additionally, it can result in a spectrum of neurodevelopmental disorders [50, 80, 101, 102, 103]. Figure 3 provides an overview of KAT6's function in physiology and pathology.

### 4.1 Cancer

In 1996, KAT6A was originally discovered as part of a chromosomal translocation, t(8; 16) (p11; p13) in the context of AML [29]. The chromosomal mutation resulted in the formation of a fusion gene, KAT6A–CBP/CREBBP, which was identified as a causal factor in leukemogenic transformations and tumorigenesis [104, 105, 106, 107, 108]. Until 2019, the KAT6A gene fusion was identified in solid tumors through next‐generation sequencing‐based targeted RNA sequencing. In a patient with renal cell carcinoma (RCC), the fusion of KAT6A with the transcription factor E3, resulting from a chromosomal translocation t(X; 8)(p11.23; p11.21), was detected [109]. The RCC case with the KAT6A–TFE3 gene fusion was observed to exhibit typical morphological features of papillae, eosinophilic cytoplasm with focal clearing, and abundant psammoma bodies, as evidenced by immunohistochemical analysis [109, 110]. These findings suggest that somatic mutations in KAT6A contribute not only to the development of hematologic malignancies but also to solid tumors.

The frequency of somatic mutations in KAT6A and KAT6B is reported to be relatively high in cancer, with 2.7% in KAT6A and 2.3% in KAT6B, respectively [34]. Aberrant expression of KAT6A and KAT6B in different types of cancer can be attributed to various prototypes of genetic alterations, including fusion, mutation, amplification, and deletion [34]. It has been reported that the abnormal expression of KAT6A or its homolog KAT6B is associated with the occurrence and development of a series of malignances [30, 31, 32, 111]. Online analyses targeting the expression of KAT6A and KAT6B between tumor tissues and paired normal tissues demonstrated that KAT6A and KAT6B were aberrantly expressed in multiple types of cancer, like RCC, breast cancer, and AML, which exhibited the most significant differences in expression profiles [34]. The dysregulation of KAT6A or KAT6B has been shown to exert a pivotal influence on survival outcomes of a multitude of cancer types, further substantiating their involvement in oncogenesis. In the majority of cases of solid tumors and hematologic malignancies, KAT6A and KAT6B function as oncogenes [31, 100, 112, 113, 114, 115, 116]. For instance, a study found that patients with high levels of KAT6B expression in TSCC tumor tissue obtained worse drug response and poorer disease‐free survival, indicating the oncogenic role of KAT6B in TSCC [59]. However, in certain cancers, they can act as tumor suppressor genes [30, 117]. Survival analysis based on online databases revealed that KAT6A was markedly, highly expressed in RCC, cholangiocarcinoma, and head and neck squamous cell carcinoma, which was associated with a favorable overall survival (OS) [34]. KAT6B upregulation was observed in patients with RCC and low‐grade glioma, and these patients harbored an extended OS [34]. In a similar trend, KAT6B inhibition in small cell lung cancer (SCLC) resulted in accelerated tumor growth, proliferation, and progression [30]. Conversely, transfection of KAT6B expression vectors into cancer cells with a homozygous KAT6B deficiency led to a reduction in tumor proliferation and a decrease in tumor size [30]. These findings infer that KAT6A and KAT6B function as tumor suppressor genes, and their upregulation is associated with improved prognostic outcomes in these tumors.

Molecular mechanisms by which KAT6 regulates tumorigenesis and drug resistance have been documented, primarily involving the PI3K/AKT signaling axis, the ER signaling pathway, and cell cycle‐related pathways (Table 1). KAT6A has been reported to modulate a suppressor at the CDKN2A locus, which inhibits cellular senescence and maintains self‐renewal of HSCs and NSCs [71, 81]. CDKN2A, which encodes p16^INK4a^ and p19^ARF^, is a multifunctional gene that plays a critical role in cell cycle and senescence [118, 119, 120]. P16^INK4A^ retains cells in G1‐phase of the cell cycle through a direct inhibition of CDK4 and cyclin D [119], while P19^ARF^ promotes senescence by enhancing the production of p53 [120]. Moreover, CDKN2A can also function as a tumor suppressor in multiple cancer types, such as melanoma, renal cancer, and pancreatic carcinoma [121, 122, 123]. Previous studies have shown that HR+/HER2− breast cancer patients with CDKN2A overexpression achieve a better response to CDK4/6i, suggesting a beneficial role for enhancing drug sensitivity [124, 125, 126]. In contrast, two specific KAT6 inhibitors have been reported to induce cell cycle exit and cellular senescence mediated by the INK4A/ARF signaling pathway, thus suppressing the tumor proliferation of hepatocellular carcinoma and lymphoma in preclinical models [36]. To see, KAT6A is capable of modulating the cell cycle through the regulation of CDKN2A, which is involved in tumorigenesis and drug sensitivity.

**TABLE 1:** Summary of KAT6 dysregulation and associated pathways in cancer.

Gene	Cancer type	Pathway	References
	ER+/HER2− breast cancer	Estrogen pathway	[32, 37, 127]
KAT6A	Lymphoma and hepatocellular carcinoma	INK4A/ARF signaling pathway	[36, 128]
	Acute myeloid leukemia	INK4A/ARF signaling pathway	[81]
	Glioblastoma	PI3K/AKT pathway	[129]
	Ovarian cancer	DNA damage repair	[130]
KAT6B	Tongue squamous cell carcinoma	PI3K/AKT/NF‐κB pathway	[59]
	Prostate cancer	PI3K/AKT pathway	[64]
	Tongue cancer	miR‐22/lncRNA/KAT6B/NF‐κB pathway	[131]

The association of KAT6 and the PI3K/AKT signaling pathway has been previously reported in prostate cancer. In Du145 prostate cancer cells, KAT6B upregulation was detected to promote tumor growth through an RNAi screening involving 44 genes involved in histone modifications. Following KAT6B knockdown, the commonly expressed‐altered genes were observed to predominantly enrich in the PI3K signaling pathway, with a reduction in its downstream p‐AKT proteins [64]. Subsequently, the role of KAT6B in the PI3K/AKT/NF‐κB signaling pathway has been documented to mediate drug sensitivity in tongue cancer cells. KAT6B was identified as a direct target of miR‐22, which has been elucidated to enhance chemosensitivity to cisplatin. Expression of KAT6B was negatively correlated with the level of miR‐22 in TSCC patients. Mechanically, miR‐22 expression and KAT6B silencing compromised the activity of PI3K/AKT/NF‐κB signaling pathway in CAL27 cell lines, through the downregulation of several cytokines, such as PDGF and VEGF [59]. The activation of the PI3K/AKT signaling pathway, regulated by the KAT6A complex, has been reported to promote tumorigenesis and the development of glioblastoma. Overexpression of KAT6A was observed across multiple glioblastoma cell lines, which enhanced cell proliferation, migration, and tumor growth, ultimately leading to worse survival outcomes. The molecular mechanism was investigated. It has been demonstrated that KAT6A upregulates PIK3CA transcription by acetylating H3K23, which recruits the oncogenic chromatin modifier TRIM24, resulting in increased AKT phosphorylation. In contrast, KAT6A knockdown reduced the levels of PIK3CA proteins and mRNA in U87 and LN229 cells, as well as the activity of downstream AKT signaling [129]. These findings infer that KAT6 functions in the development of tumorigenesis and drug resistance via the regulation of the PI3K/AKT signaling pathway.

The role of KAT6 in the ER signaling pathway in breast cancer has been extensively investigated. Through acetylating H3K23, KAT6A regulates the activity of the ER signaling pathway by mediating ESR1 at both transcriptional and translational levels [127, 132]. However, the specific molecular mechanisms by which KAT6 regulates ER signaling remain unexplored. It has been shown that ER+/HER2− breast cancer patients with PIK3CA mutation exhibited worse survival benefits from KAT6 inhibitor‐based therapy [37]. Given the close crosstalk between the PI3K/AKT and ER pathways [133, 134], this may represent a key direction for future basic research. Furthermore, in ER+/HER2− breast cancer following progression on CDK4/6i, genetic alterations associated with KAT6A inhibition were found to be enriched in cell cycle and associated pathways [37], such as the E2F pathway. This also provides insights for future laboratory investigations.

In addition, a recent study has shown that KAT6A knockdown can restore drug sensitivity to poly(ADP‐ribose)‐polymerase (PARP) inhibitor (PARPi) in ovarian cancer cells that develop drug resistance to PARPi [130]. The molecular mechanism has been explored. In PARPi‐resistant ovarian cancer cells, the liquid–liquid phase separation (LLPS) of KAT6A reduced the cytotoxic effects of PARPi treatment by releasing PARP1 trapped at the DNA break sites. However, using the KAT6 inhibitor to block the process of LLPS, PARPi sensitivity was detected to be enhanced by inhibiting DNA damage repair in ovarian cancer in vivo and in vitro [130]. It has been widely accepted that BRCA1/2 mutations or homologous recombination deficiency contribute to survival benefits from PARPi treatment [135, 136, 137]. Therefore, the correlation of KAT6A and BRCA1/2 mutations or homologous recombination deficiency remains to be investigated.

To conclude, both KAT6A and KAT6B have a crucial impact on the tumor proliferation, metastasis, and drug resistance of multiple types of cancers, via regulating the PI3K/AKT signaling axis, the ER signaling pathway, and cell cycle‐related pathways.

### 4.2 Neurodevelopmental and Neurological Disorders

As previously mentioned, KAT6 plays a critical role in tissue development, and its dysregulation is expected to be associated with neurodevelopmental abnormalities and disorders. Indeed, germline mutations in KAT6A have been reported to link to neurodevelopmental disease called KAT6A syndrome or Arboleda‐Tham syndrome (OMIM #616268), while KAT6B is mainly associated with genitopatellar syndrome (GPS) (OMIM #606170) and Say–Barber–Biesecker–Young–Simpson Syndrome (SBBYSS) (OMIM #603736) [138, 139].

#### 4.2.1 KAT6A Syndrome

As a genetic congenital disorder, KAT6A syndrome is an autosomal dominant hereditary disease [140, 141, 142, 143, 144, 145, 146, 147]. Mutations in the KAT6A gene serve as an important cause of KAT6A syndrome that is also referred to as Arboleda‐Tham syndrome. KAT6A mutations are frequently observed in the acidic domain, rather than in the MYST domain. Truncating mutants and nonsense mutations exhibit as the primary mutation type [140, 141, 142]. Truncated frameshifts are related to severe symptoms [79]. Hot spots of nonsense mutations make up approximately 20% of patients with KAT6A syndrome, which are located at amino acid positions 1019, 1024, and 1129 in the acidic domain [41, 79]. Missense mutations and changes in the splice site have also been reported in some real‐world patients [79].

In general, KAT6A syndrome manifests intellectual impairment, language deficits, and global developmental delay [140, 141, 142]. Other features, including speech delay, primary microcephaly, recurrent infection, craniosynostosis, neonatal hypotonia, feeding difficulties, digestive complications, and sleep disorders, expand the clinical spectrum of this syndrome [41, 143]. An emerging study indicated that the absence of BRPF1 within KAT6 complexes may be the molecular mechanism. Elaborately, BRPF1 knockdown resulted in a reduction in the frequency of miniature excitatory postsynaptic currents and a downregulation of gene expression implicated in neural development, synapse function, and memory. These genes included Pcdhgb1, Slc16a7, Robo3, and Rho [148].

Additionally, due to the advancement of modern technology, such as whole‐exome sequencing, pathological variants of KAT6A have been identified in a broader spectrum of patient cases. They include syndromic craniosynostosis, epilepsy, pituitary stalk interruption syndrome, and congenital neutropenia [149, 150, 151, 152].

#### 4.2.2 KAT6B‐Associated Neurological Disorders

KAT6B mutations have been shown to predominantly correlate with GPS and SBBYSS, as well as other phenotypic spectra of neurodevelopmental syndromes, such as Noonan syndrome (OMIM #163950), and syndromic craniosynostosis [103, 153, 154, 155, 156, 157, 158].

GPS and SBBYSS are caused by distinct mutations in the KAT6B gene, which serve as rare autosomal dominant disorders with neurodevelopmental abnormalities [103, 159]. KAT6B variants are primarily de novo dominant mutations, leading to truncated frameshift. Other types, including in‐frame deletions and missense mutations have also been identified in patients [101, 103]. Mutations associated with GPS are located in the proximal portion of the last exon, which results in the expression of a protein without a C‐terminal domain. Mutations associated with SBBYSS occur throughout the gene or more distally in the last exon, among which the former mutation type leads to nonsense‐mediated decay [153, 159]. Most mutations have been reported to occur in exon 18, with a few cases in exon 14 [160].

Regarding clinical manifestations, both GPS and SBBYSS present with overall severe intellectual disability, congenital heart defects, hypotonia, and patellar anomalies [80, 159, 161]. There are differences between the two syndromes. GPS is featured by developmental abnormalities in the patella and the knee joint, such as contractures. Some may display a structural defect in the brain, such as corpus callosum detachment. And patients with SBBYSS have long thumbs and long great toes, lacrimal duct abnormalities, as well as characteristic facial features [80, 102, 158, 159, 160, 161, 162]. Molecular mechanisms are mainly associated with dysregulation of histone acetylation from specific KAT6B mutations. In mice models, SBBYSS‐specific KAT6B deficiency resulted in learning and social deficits, through a potential mechanism of a reduction in H3K9Ac [163]. Moreover, in GPS patient‐derived cells harboring a heterozygous truncating mutation in KAT6B, a reduction in the global acetylation of H3 and H4 has been observed [153].

Noonan syndrome is associated with a translocation that interrupts intron 3 of KAT6B, which can result in KAT6B haploinsufficiency and a reduction of global H3 acetylation [164]. The syndrome shares some common clinical features with GPS and SBBYSS, involving facial dysmorphism, developmental delay in growth and height, cardiac abnormalities, skeletal malformations, and cognitive defects [165, 166]. The RAS/MAPK pathway has been shown to be implicated in Noonan syndrome [164, 165, 166]. Specifically, such KAT6B alternation enhanced phosphorylation of MEK1/2, ERK, and AKT, which could be reversed when transfecting a high‐expressing KAT6B construct to cells derived from patients with Noonan syndrome [166]. Moreover, in KAT6B knockdown cell lines and KAT6B‐deficient mouse model, a wide range of genes related to the MAPK signaling pathway have been revealed to downregulate [166]. These findings suggest the correlation between the RAS/MAPK pathway and Noonan syndrome.

In conclusion, KAT6 mutations are capable of leading to multiple types of neurodevelopmental disorders, like KAT6A syndrome, GPS, SBBYSS, Noonan syndrome, and so on.

### 4.3 Other Diseases

Beyond its associations with tumorigenesis and neurodevelopmental delays, KAT6 aberrations are also implicated in a spectrum of other developmental disorders, among which craniofacial dysmorphism and skeletal abnormalities are the most frequently reported. This abnormality typically co‐occurs with neurodevelopmental delays, rarely presenting in isolation.

Both KAT6A‐ or KAT6B‐deficiency and LoF mutations have been shown to lead to facial dysmorphogenesis and cleft palate in some patients, but the specific condition differs to some extent [23, 66, 167]. Specifically, facial dysmorphogenesis features common to KAT6A patients embrace subtle facial features such as bi‐temporal narrowing, broad nasal tip, thin upper lip, posteriorly rotated or low‐set ears and micro/retrognathia. Molecular mechanisms underlying such a condition have been demonstrated. In mice models, the downregulation of KAT6A affects the expression of several genes associated with the formation of cleft palate, including Dlx1, Dlx2, Dlx3, Dlx4, and Dlx5 [167, 168, 169, 170]. The Dlx homeobox gene family regulates embryonic development, and Dlx proteins regulate transcription for bone development of the jaws and limbs. Besides, the downregulation of KAT6A in pharyngeal arches and palatal shelves leads to elevated expression of transcription factors that participated in osteoblast differentiation, including Runx2, Pax9, and Nkx3‐1 [167, 171, 172].

In contrast, KAT6B patients display low‐set and posteriorly rotated ears, bulbous nose, flat broad nasal bridge, prominent cheeks, thin upper lip, and micro/retrognathia. Some patients with KAT6B mutations exhibit unique facial features. For instance, SBBYSS is characterized by an immobile mask‐like face, with a series of features like ptosis, lacrimal canal stenosis, and generally long fingers and toes. A subset of GPS patients has been observed with flexion contracture of the hip joint, microcephaly, and craniofacial dysmorphism. In addition, spinal developmental defects, like congenital scoliosis, have also been reported in patients with KAT6B alterations, which is probably caused by the downregulation of KAT6B mediated by trimethylation of H3K27 on the KAT6B promoter [167, 173].

To conclude, aberrations of KAT6A and KAT6B are correlated with cancers, distinct neurodevelopmental syndromes, together with other developmental diseases like craniofacial dysmorphism and skeletal abnormalities.

## 5 Therapeutic Targeting of KAT6

Recently, the therapeutic effect of inhibiting KA6 has achieved a milestone in the field of breast cancer. It was first reported that KAT6 inhibitor‐combined therapy demonstrated a manageable safety profile and profound antitumor efficacy in patients with ER+/HER2− ABC patients in a phase 1 dose escalation and dose expansion study [37].The study has significant implications for further research in both scientific and clinical fields on the targeting of such new epigenetic machinery. Herein, we mainly discuss KAT6 as a therapeutic target for breast cancer due to its potential as a druggable target and the necessity of a systematic review which has not been summarized and reported in the previous literature.

### 5.1 KAT6 as a Biomarker

As previously noted, KAT6 can function either as a tumor suppressor or proto‐oncogene across various cancer types, implicated in tumor initiation and progression. Proto‐oncogenes that are frequently overexpressed and associated with poor prognosis—such as KAT6A in breast cancer—are generally considered attractive therapeutic targets or clinically actionable biomarkers [100]. In contrast, tumor suppressors that exhibit high expression correlated with favorable outcomes, or low expression linked to poor prognosis, may not align with conventional drug development paradigms. Examples include KAT6A, which acts as a tumor suppressor in RCC, head and neck squamous cell carcinoma, and SCLC [34].

Metagenomic analysis of KATs in human cancers revealed that KAT6A obtained high frequencies of genomic amplification or mutation in breast cancer [174]. KAT6A is situated within the 8p11‐12 amplicon, exhibiting a high prevalence of amplification, with a frequency range of 10–15% in breast cancer patients [32, 100]. The amplification of KAT6A was correlated with an unfavorable prognosis in patients with ER+/HER2− breast cancer [32, 100]. Regarding transcriptional aspects, KAT6A mRNA has been observed to be overexpressed in ER+ breast cancer, accounting for about 15% of breast cancers and a higher percentage of 22% in ER+/HER2− breast cancer [32]. KAT6A overexpression is highly correlated with gene amplification [32], suggesting their similar roles in clinical outcomes of ER+/HER2− breast cancer. High expression of KAT6A has been shown to result in poor survival outcomes in ER+ breast cancer [32, 34, 175], indicating that KAT6A functions as an oncogene in ER+/HER2− breast cancer.

The molecular mechanism underlying the oncogenic role of KAT6A in ER+/HER2− breast cancer has been explored. KAT6A has been reported as a direct transcriptional mediator of ESR1 which encodes ER‐alpha (ERα) [32]. As a principal regulator of the hormone pathway, dysregulation of ERα drives tumorigenesis and endocrine resistance of ER+ breast cancer [176, 177, 178, 179]. An increase in KAT6A levels enhances ESR1 mRNA and ERα expression, which in turn is linked to more aggressive behaviors of ER+/HER2− breast cancer cells. In KAT6A‐overexpressed ER+/HER2− breast cancer models, KAT6A was found to bind to the promoter of ERα and increase its expression, thereby promoting tumor cell proliferation [180]. Conversely, KAT6A knockdown or inhibition attenuates breast cancer cell growth and exerts antitumor effects, encompassing a mechanism of eradicating ERα expression at the transcriptional level.

In addition, a newly published study has shown that KAT6A can serve as a druggable marker in NUP98‐rearranged AML [181]. Through rapid immunoprecipitation mass spectrometry of endogenous proteins, interaction between NUP98 fusion oncoprotein and MYST HAT complexes, including KAT6A and KAT6B, was observed in eight preclinical models of NUP98‐rearranged AML. Further analyses revealed that genes involved in histone acetylation play a pivotal role in the development of AML, which is consistent with the colocation of the KAT6 and NUP98 fusion oncoproteins. Knockdown of MYST family HAT complex members, including KAT6A and KAT6B, leads to reduced cell fitness in AML [181]. These findings indicate KAT6 may play a central role in NUP98‐r leukemogenesis, which can serve as a biomarker for ensuing pharmacologic inhibition.

To see, KAT6A and KAT6B can function as biomarkers for malignancies, exerting great potential to develop as pharmacological targets.

### 5.2 Pharmacological Inhibition

Recently, the mechanistic basis for pharmacological inhibition of KAT6 has been comprehensively delineated. In ER+/HER2− breast cancer cell lines that highly express KAT6A, pharmacological inhibition of KAT6A resulted in a dose‐dependent decrease in ESR1 mRNA and ERα protein expression, and weakened tumor cell proliferation and invasion. Moreover, in ER+ organoid models from human tumors and patient‐derived xenografts (PDXs), the administration of KAT6 inhibitor downregulated genes that were enriched for early and late estrogen response. To be more elucidated, the changes in estrogen were accompanied by a loss of H3K23Ac [180], indicating the epigenetic molecular mechanism of KAT6A inhibition for antitumor activity in breast cancer. KAT6A enzymes catalyze H3K23Ac at both global and locus‐specific levels, thus KAT6A inhibition is anticipated to result in a decrease or loss of H3K23Ac. The process can be observed in both sensitive ER+ and resistant ER− cell lines, but only the reduction of H3K23Ac in ER+ breast cancer cells led to antitumor efficacy [132]. This implies similar target inhibition but selective requirement for KAT6 in ER+ breast cancer cells only. An assay for transposase‐accessible chromatin with high‐throughput sequencing demonstrated that the KAT6 inhibitor regulated KAT6A and KAT6B acetyltransferase activity by directly inhibiting the recruitment of RNA Pol II to the ESR1 promoter, thus reducing chromatin accessibility for ESR1 transcription and H3K23Ac. In normal cells and MCF7 cell lines with low KAT6A expression, minimal KAT6A downregulation together with limited ESR1 inhibition were observed, even in the presence of global loss of H3K23Ac [180]. These findings suggest that transcriptional inhibition of ESR1, as well as overexpression of KAT6A, are presumably determinants of drug response to KAT6 inhibitors in ER+ breast cancer.

However, another emerging study showed that drug sensitivity to the KAT6 inhibitor was mediated by ER expression in ER+/HER2− breast cancer models, rather than KAT6A protein levels [132]. Further dose titration experiments showed that the combination of the selective ER downregulators (SERDs), such as fulvestrant and elacestrant, with the KAT6 inhibitor contributed to synergistic effects in MCF7 and T47D breast cancer cells, increasing their sensitivity to ET. Furthermore, in the PDX model derived from a breast cancer patient who develops drug resistance to tamoxifen, letrozole, and fulvestrant, KAT6 inhibitor‐combined therapy demonstrated robust tumor shrinkage, suggesting its role in ET‐resistant settings. These findings suggest that ER signaling is responsible for the pharmacological inhibition of the KAT6 inhibitor, and a combination strategy involving the KAT6 inhibitor may potentially reverse endocrine resistance. Although studies on KAT6 inhibition reveal differences, they suggest that KAT6 inhibitors are suitable for patients with ER+ breast cancer, and combining KAT6 inhibitors with ET may achieve enhanced clinical benefits.

For ER+ breast cancer cell lines, which were sensitive to KAT6 inhibitors, the activity of multiple signaling pathways associated with tumor growth was inhibited, including ER signaling, the cell cycle, and the E2F pathway, as well as luminal transcription factors and coactivators such as ESR1, PGR, E2F, and MYC [180]. Further analysis of these genes, which were downregulated by the KAT6 inhibitor, revealed that they were primarily involved in estrogen signaling, the E2F pathway, MYC signaling, the cell cycle, and epithelial–mesenchymal transition [180]. What intrigues us is the downregulation of the E2F pathway. The activation of the CDK–RB–E2F axis, which promotes the G1/S phase transition, regulates the cell cycle at the molecular level. CDK4/6 inhibition depends on the intact cyclin D–CDK4/6–RB1–E2F axis [182]. KAT6 inhibition leads to a decrease in E2F transcription and the activity of the E2F pathway, suggesting a possible synergistic effect of KAT6 inhibitors combined with CDK4/6i, and perhaps the reversal of CDK4/6i resistance. Another piece of evidence that supports this hypothesis is the fact that KAT6A and another oncogene FGFR1 are located on the chromosome in the same position (chromosome 8p11‐p12) [100, 183, 184], indicating their analogous functions. FGFR1 amplification occurs in 15% of ER+ breast cancers, which has been shown to correlate with resistance to CDK4/6i and ET [185]. Therefore, it is worth exploring whether KAT6A amplification and overexpression can cause drug resistance to CDK4/6i.

Due to the paralogous structure of KAT6A and KAT6B, most currently available KAT6 inhibitors are capable of concurrently inhibiting both KAT6A and KAT6B. However, it seems that the antitumor effects were demonstrated through the predominant inhibition of KAT6A and a comparatively limited restriction of KAT6B. In ER+/HER2− breast cancer cell models, a significant correlation was observed between the cell line sensitivity (AUC) of a KAT6‐specific inhibitor and the BRPF1 dependency score of KAT6A as compared with KAT6B [180]. A few ER+/HER2− breast cancer cells overexpressing KAT6B showed inhibitory activity against the KAT6‐specific inhibitor, but no significant correlation was observed between KAT6B expression and drug sensitivity. These findings suggest that KAT6A inhibition leads to dominant antitumor effects on breast tumors, while KAT6B inhibition may have a potential pharmacological role. Data on its homolog, KAT6B, in breast cancer are relatively limited. A study showed that KAT6B knockdown in breast cancer cell lines decreased cellular proliferation and migration of tumor cells and prompted G_2_/M cell cycle arrest. Further microarray analyses following KAT6B knockdown revealed a reduction in the expression of a series of genes associated with tumor growth and invasiveness [186], which indicates the oncogenic impact of KAT6B in breast cancer. However, emerging evidence has shown that only the concurrent suppression of KAT6A and KAT6B exerts profound antiproliferative effects in MCF‐7 and MDA‐MB‐361 cells, whereas inactivation of KAT6A achieved efficacy comparable to the dual inactivation of both KAT6A and KAT6B in T47D cells [132]. These findings suggest that KAT6A remains the primary target for pharmacological inhibition of KAT6, particularly in ER+ breast cancer.

To sum up, although most KAT6 inhibitors exhibit dual pharmacological activity against both KAT6A and KAT6B, their antitumor effects are predominantly mediated through the pharmacological inhibition of KAT6A. The therapeutic potential of targeting KAT6B remains to be further elucidated.

### 5.3 Emerging Strategies

Targeting the acetylation pathway represents a promising epigenetics‐oriented approach for the treatment and reversal of drug resistance in malignancies, including BRD reader inhibitors (such as BET inhibitors) and HDAC inhibitors [187, 188, 189, 190, 191]. Some epigenetic drugs have shown meaningful efficacy in the treatment of hematologic malignancies, which are currently being prepared for clinical approval [187]. Nevertheless, the development of epigenetic drugs has not been as significant in treating solid tumors due to the limited and variable therapeutic effects observed. To illustrate, in reported phase 3 clinical trials, HDAC inhibitors, including entinostat and chidamide, have yielded disparate survival outcomes in HR+/HER2− ABC patients who have experienced resistance to ET [192, 193]. In the pivotal E2112 clinical trial, entinostat in combination with ET resulted in a median progression‐free survival (PFS) of 3.3 months, which was not statistically significant when compared with ET alone [192]. Another clinical trial, designated ACE, demonstrated that the addition of chidamide to ET contributed to a notable improvement in PFS, from 3.8 to 7.4 months, in patients with HR+/HER2− ABC [193]. Further investigation is necessary in future clinical trials to ascertain whether HDAC inhibitors can be employed as a treatment strategy for patients with HR+/HER2− ABC.

**FIGURE 4:**
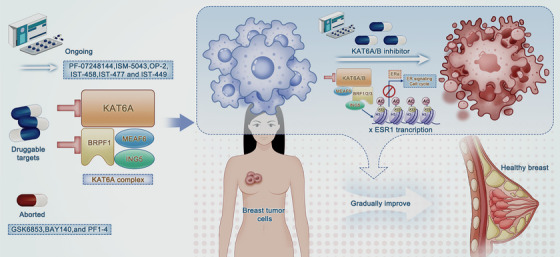
Druggable targets of the KAT6 protein and KAT6 inhibition in breast cancer. KAT6A protein is comprised of KAT6A, BRPF1, ING5, and MEAF6, among which KAT6A and BRPF1 have been identified as potential antitumor targets for drug discovery. Drugs targeting BRPF1 failed due to incomplete enzymatic suppression and limited antitumor effects. Currently, available KAT6 enzymatic inhibitors primarily focus on dual suppression of KAT6A and KAT6B activity, involving PF‐07248144, ISM‐5043, OP‐2, IST‐458, IST‐477, and IST‐449. These small‐molecule KAT6 modifiers decrease ERα expression via direct inhibition of ESR1 transcription, which is frequently accompanied by a reduction in H3K23Ac. Subsequently, the accumulative effects of KAT6 inhibition could exert an antitumor role in breast cancer via the suppression of ER signaling transduction as well as the cell cycle.

Recently, an epigenetic modifier targeting KAT6 combined with ET has shown encouraging efficacy in preclinical models and a phase 1 clinical trial [37, 180], highlighting the potential of KAT6 inhibitors for the treatment of HR+/HER2− ABC. Although KAT6 inhibitors have made remarkable progress in the treatment of solid tumors, the process for discovering drugs that inhibit KAT6 has encountered a broad range of challenges along the way (Figure 4). In the early stages of research into KAT6 inhibitors, the identification of small molecule ligands for BRD situated within the BRPF1 protein was accomplished, leading to the discovery of GSK6853, BAY140, and PFI‐4 [194, 195, 196]. These targeted approaches partially affect the function of the KAT6 complex by inhibiting BRD protein–protein interactions, thereby restraining malignancy. However, they fail to result in genetic knockout or enzymatic suppression [197], indicating limited antitumor effects. Additional molecular mechanisms that influence the therapeutic effects of these inhibitors include BRPF1 splicing isoforms, insertion of residues in BRD, and differential expression of BRPF1a and the targetable BRPF1b isoform among tissues [195, 198].

The year 2018 saw the discovery of selective inhibitors targeting the KAT6 enzyme, marking a milestone in the development of KAT6 inhibitors. This was linked to a competitive compound targeting KAT6A, designated CTx‐0124143, which was derived from a high‐throughput screening (HTS) procedure [199]. Based on the lead compound of CTx‐0124143, the acylsulfonohydrazide WM‐1119, together with benzoylsulfonohydrazides WM‐8014 and WM‐3835, have been synthesized [36, 128]. These KAT6 inhibitors were shown to exhibit effective inhibition against both KAT6A and KAT6B, induce cellular senescence, and impede tumor growth in models of MYC‐driven lymphoma and AML [36]. However, their pharmacological efficacy is constrained by several unfavorable drug properties, including off‐target effects, poor pharmacokinetics (PK), inadequate absorption, variable serum concentration, and rapid metabolism. The identification of new chemical molecules with reformative drug‐like properties compared with sulfonohydrazide‐based compounds is required to fully leverage the therapeutic potential of KAT6A inhibition.

In recent years, there has been a significant investment in research and development focused on KAT6 enzymatic inhibitors, resulting in the creation of novel compounds with enhanced affinity for both KAT6A and KAT6B. These novel small‐molecule inhibitors have been optimized with respect to their physicochemical, PK, absorption, distribution, metabolism, and excretion (ADME) properties. In theory, the improved drug‐like properties can augment inhibitory effects while concurrently reducing AEs, thereby rendering them more tolerable and applicable in clinical practice. This is particularly evident in the field of breast cancer research, where a series of KAT6‐targeting inhibitors have demonstrated robust antitumor efficacy and a manageable toxicity profile both in preclinical studies and clinical trials [37, 132, 200, 201, 202] (Figure 4).

**FIGURE 5:**
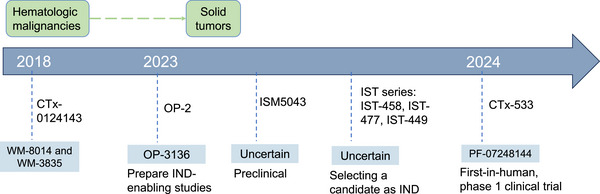
Drug development of targeted inhibitors focuses on KAT6. From 2018 to 2024, KAT6 enzymatic inhibitors have made significant progress, especially in solid tumors. The milestone for its drug development is that PF‐07248144 achieved manageable toxicity and sustained antitumor activity in breast cancer in the first‐in‐human clinical trial.

In the following sections, we collect and summarize the currently available information on KAT6 inhibitors that have the potential to be therapeutically beneficial in the arena of ER+ breast cancer (Table 2). As the inaugural benzisoxazole series to be investigated in human trials, PF‐07248144 has demonstrated considerable promise in managing patients with ER+/HER2− ABC. Other KAT6 inhibitors are showing favorable PK and safety profiles in preclinical cell lines and animal models of ER+/HER2− breast cancer [200, 201, 202, 203, 204], which may offer a promising avenue for entering in‐human clinical trials and provide a therapeutic choice for these patients. Figure 5 shows an overview of the development of KAT6 inhibitors in chronological order.

**TABLE 2:** Current available KAT6 catalytic inhibitors in ER+/HER2− breast cancer.

Compound	Chemical structure	Progress	Combinations
PF‐07248144 [37, 205]	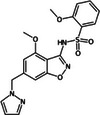	Phase III clinical trial (recruiting, NCT04606446)	Fulvestrant
IST‐458, IST‐477 and IST‐449 [200, 201]	Not available	Selecting a candidate for IND‐enabling studies	Monotherapy
ISM5043 [202]	Not available	Preclinical	Tamoxifen Fulvestrant
OP‐3136 [203]	Not available	Phase I clinical trial (recruiting, NCT06784193)	Palazestrant CDK4/6i

*Abbreviation*: IND: investigational new drug.

### 5.4 Preclinical and Clinical Advances

#### 5.4.1 CTx‐648 (PF‐9363) and PF‐07248144

Currently, PF‐07248144 remains the only KAT6 inhibitor to have entered clinical trials for humans. Its emergence has notably bolstered scientific confidence in epigenetic therapeutics. The compound has undergone a complex evolution from its initial precursor, CTx‐648, to the current clinical agent, PF‐07248144.

CTx‐648 (also known as PF‐9363) is a type of benzisoxazole sulfonamide compound, with a 6‐position benzisoxazole core as the 6‐(1H‐pyrazol‐1‐yl) methyl) substituted molecule, which binds to the acetyl‐CoA binding site of KAT6A catalytic to exert its pharmacological effects [127, 180]. The discovery of CTx‐648 was initiated by an HTS campaign that explored a diversity library of 250,000 compounds. This campaign selected a competitive molecule of CTx‐533 that directly binds to KAT6A, which was subsequently identified as the lead benzisoxazole compound. Through medicinal chemistry lead optimization, new analogs of CTx‐648 were generated on the basis of the original HTS hit CTx‐533. CTx‐648 new compounds displayed improved potency, with an IC50 of 0.37 nM against cell proliferation and an IC50 of 0.85 nM against H3K23Ac pertaining to the MYST family. Data from in vivo assessment showed that CTx‐648 had over a 1800‐fold improvement in KAT6A affinity compared with the initial compound CTx‐533, which resulted in more than an 11,000‐fold enhancement in potency toward H3K23Ac [180]. Additionally, CTx‐648 displayed more drug‐like properties, robust hepatocyte stability, low intrinsic clearance, and excellent oral bioavailability in animal testing on rodents. In ER+/HER2− cell lines and breast cancer PDX models, CTx‐648 demonstrated potent and selective inhibitory activity against the KAT domains of KAT6A, yielding strong and tolerable on‐target in vivo efficacy involving tumor regression with minimal toxicity [180]. Recently, treatment combining CTx‐648 has been shown to demonstrate robust efficacy in various ET‐resistant ER+/HER2− breast cancer cell models, involving doxycycline‐inducible HA‐ESR1 Y537S models, NF1‐deficient models, and FOXA1 F266L and SY242CS mutants in T47D and MCF7 cells [132]. It has been widely recognized that the most common mechanisms of acquired endocrine resistance are mutations in ESR1, alterations in transcription factors such as FOXA1 and MYC, and dysregulation of signaling pathways, like NF1 deficiency or HER2 amplifications [206, 207, 208]. These findings provide preclinical proof of concept that KAT6A is a druggable cancer target, supporting the notion that a novel class of epigenetic drugs targeting KAT6A may be a practical and effective approach for ER+/HER2− breast cancer. Based on the favorable properties of the benzisoxazole‐based compound, a structurally related molecular entity, namely, PF‐07248144, was synthesized and subsequently investigated in a phase 1 clinical trial for the treatment of metastatic solid tumors (ClinicalTrials.gov Identifier: NCT04606446) [37, 209].

*Nature Medicine* has recently presented the preliminary results of the first‐in‐human, phase 1 dose‐escalation and dose‐expansion study of PF‐07248144 [37]. In the dose escalation phase, the recommended dose for expansion (RDE) of 5 mg once daily for PF‐07248144, either as monotherapy or in combination with 500 mg of fulvestrant, was identified using a Bayesian logistic regression model. The maximum tolerated dose for PF‐07248144 was not reached, suggesting that the drug has minimal toxicity and is clinically safe. Further dose expansion part included 43 patients with ER+/HER2− ABC to receive PF‐07248144 5 mg q.d. combined with fulvestrant 500 mg, and 35 patients to receive PF‐07248144 as monotherapy. All patients have received CDK4/6i prior to the initiation of PF‐07248144‐based therapy. Stratified by prior lines of systemic therapies, 23 patients were assigned to receive PF‐07248144 plus fulvestrant as second‐line (2L) therapy, and 20 patients received the combination therapy as third‐line and the above (3L/3L+) treatment.

Antitumor activity of PF‐07248144, involving objective response rate (ORR), duration of response (DOR), clinical benefit rate (CBR), and PFS, was evaluated [37]. In the combination group (*n* = 43), 13 patients achieved partial response, leading to an ORR of 30.2% (95%CI: 17.2–46.1%). The median PFS was reported as 10.7 months (95%CI: 5.3–NE months), which is evidently longer than the 3.3 months (95%CI: 2.0–5.8 months) observed in patients who received PF‐07248144 alone. Other results were also promising, with the median DOR of 9.2 months (95%CI: 7.2–NE months), and the CBR of 51.2% (95%CI: 35.5–66.7%). For patients in the 2L group (*n* = 23), the ORR was reported as 21.7% (95%CI: 7.5–43.7%) and the CBR was 43.5% (95%CI: 23.2–65.5%). The median PFS was not mature. For patients in the 3L/3L+ group (*n* = 20), the ORR was calculated as 40.0% (95%CI: 19.1–63.9%) and the CBR was 60.0% (95%CI: 36.1–80.9%). The median PFS was observed to be 10.7 months (95%CI: 5.5–NE months). To see, the combination of PF‐07248144 and ET contributed to meaningful clinical benefits for patients with ER+/HER2− ABC in the post‐CDK4/6i setting.

With regard to the safety profile, it was determined that AE resulting from the combination of PF‐07248144 and fulvestrant therapy could be effectively managed. The most common AE of any grade was dysgeusia, accounting for 86.0% among all patients, with no instances of grade 3 (G3) or above. This phenomenon can be explained by the fact that epigenetic changes in transcriptional factors or genes have an impact on taste perception [210]. KAT6A serves as a critical epigenetic regulator for posttranslational modifications of histones, which physically relaxes the chromatin and activates transcription [15]. In theory, KAT6A inhibition by PF‐07248144 can recover a compact and ordered chromatin structure that interferes with the activity of transcriptional factors associated with taste, thereby resulting in altered taste or dysgeusia. Other AEs, including neutropenia (28 out of 43, 65.1%), fatigue (24 out of 43, 55.8%), and anemia (19 out of 43, 44.2%), represented a relatively high percentage. Treatment‐related AEs (TRAEs) ≥ G3 primarily manifested as myelosuppression, such as neutropenia [G3: 18 cases (41.9%), G4: one case (2.3%)], leukopenia [G3: five cases (11.6%)], and anemia [G3: four cases (9.3%)]. Neutropenia or other TRAEs could be alleviated or reversed through dose reduction [37], suggesting that these TRAEs come from toxicity associated with KAT6 inhibition, rather than the activation of the immune system.

In conclusion, PF‐07248144, a selective catalytic KAT6A and KAT6B inhibitor, exhibits a manageable safety profile and durable antitumor activity in patients with ER+/HER2− ABC who have progressed after CDK4/6i plus ET in the pivotal phase 1 clinical trial. At present, CDK4/6i combined with ET represents the optimal first‐line treatment for patients with ER+/HER2− advanced or metastatic breast cancer [211, 212]. Considering that PF‐07248144 plus ET displays encouraging antitumor effects, this new combination regimen may emerge as a preferred option for patients with ER+/HER2− ABC following CDK4/6i treatment. Recently, a phase III clinical trial has been planned in such a patient population, which allocated patients with 5 mg QD PF‐07248144 plus fulvestrant treatment versus fulvestrant alone [205]. It is anticipated to further demonstrate the efficacy and safety of KAT6 inhibitor‐based treatment strategy in patients with ER+/HER2− ABC after progression on CDK4/6i plus ET.

#### 5.4.2 Isosterix KAT6 Inhibitors

In 2023, at the San Antonio Breast Cancer Symposium, two structurally distinct inhibitor series (series 1 and series 2) with profound antitumor activity in xenograft models were reported [200]. Concerning selectivity for KAT6 complexes, these compounds possessed superior specificity for KAT6A (IC50 < 5 nM) and limited selectivity against KAT6B (IC50 > 500 nM). At some level, this represents progress in selectivity, given the wide range of functional differences between KAT6A and KAT6B.

Preclinical data revealed that these lead compounds had promising PK parameters, tolerated toxicity, low intrinsic clearance, and good oral bioavailability. Based on their favorable properties, medicinal chemistry lead optimization resulted in the development of candidates from both series. Series 1 included IST‐458, and series 2 included IST‐477 and IST‐449. They induced tumor growth suppression and reduced cell viability in ER+/HER2− breast cancer cells overexpressing KAT6A, with IST‐477 exhibiting the most pronounced inhibitory effects. In an ER+/HER2− mouse xenograft model, KAT6 inhibitors, including IST‐458 p.o. QD and IST‐449 p.o. BID, displayed dose‐dependent antitumor activity as monotherapy. Further mechanistic explorations showed that treatment with KAT6 inhibitors led to dose‐dependent cell cycle arrest and senescence, as well as apoptosis in breast tumor cells, which was generally similar to previous studies [36, 37, 180]. IND‐enabling studies with a single development candidate were reported to be planned in the prospective future [200].

#### 5.4.3 ISM5043

The compound ISM5043 presents the discovery of KAT6 inhibitors with robust antitumor effects in both in vitro and in vivo settings. It demonstrated potent selectivity for KAT6A and KAT6B versus other KAT family members of KAT5, KAT7, KAT8, P300, CBP, and so on, with an IC50 of 5 nM against KAT6A and an IC50 of 10 nM against KAT6B. In KAT6A‐amplified ER+/HER2− breast cancer cell lines, including ZR‐75‐1 and CAMA‐1, ISM5043 demonstrated dose‐dependent antitumor efficacy as monotherapy, which correlated with the suppression of H3K23Ac and ERα protein levels. In vitro, ISM5043 displayed favorable ADME properties as well as desirable PK and safety profiles, characterized by slow clearance with a half‐time of more than 4 h, low risks for CYP inhibition or induction, good kinetic solubility, and metabolic stability. In ER+/HER2− breast cancer xenograft mouse models, the combination of ISM5043 with either tamoxifen or fulvestrant demonstrated a synergistic effect on tumor growth. Furthermore, in a PDX model derived from an ER+/HER2− breast cancer patient who progressed after multiple prior lines of chemotherapy and ET (including CDK4/6i palbociclib plus letrozole), ISM5043 as monotherapy greatly inhibited tumor growth. It suggests that ISM5043 may be developed as a promising therapeutic option for ER+/HER2− breast cancer patients who experienced disease progression following CDK4/6i‐based therapy. The evidence provides a theoretical basis for the efficacy of monotherapy with ISM5043 or combination therapy with other agents for ER+ breast cancer. The manufacturer is currently engaged in further research to propose the use of ISM5043 in the treatment of both solid tumors and hematologic malignancies.

#### 5.4.4 OP‐2

OP compounds (OP‐1 to OP‐5), as novel KAT6 inhibitors, demonstrated selective KAT6‐targeting effects, with OP‐2 representing the only compound to have an IC50 lower than 10 nM against both KAT6A and KAT6B. What is worth mentioning, OP‐2, along with other OP compounds, exhibited potentiated selectivity against KAT6B (as evidenced by biochemical assays), which differentiates it from other KAT6 inhibitors being investigated in the context of breast cancer. As previously mentioned, current evidence supports KAT6A as an oncogene in ER+/HER2− breast cancer, while the oncogenic role of KAT6B remains less clearly elucidated. Existing KAT6 inhibitors primarily target KAT6A, with limited efficacy against KAT6B. Given their distinct physiological functions, such as modulation of TME, it warrants careful consideration whether simultaneous potent inhibition of both KAT6A and KAT6B would yield the desired antitumor effects.

Based on recently released preclinical data, the OP compounds, which potently inhibit both KAT6A and KAT6B, exhibit robust antitumor efficacy in ER+/HER2− breast cancer. Among all OP compounds, three types, specifically OP‐2, ‐4, and ‐5, have demonstrated remarkable antitumor efficacy in both in vitro and in vivo models. In multiple ER+/HER2− breast cancer cell lines with KAT6A overexpression, the compounds exhibited antiproliferative effects in accordance with inhibition of H3K23Ac and ERα expression. This phenomenon was observed in breast cancer cell lines with ESR1 mutations. In an in vivo ZR‐75‐1 breast cancer xenograft model, OP‐2, ‐4, and ‐5, resulted in dose‐dependent tumor growth inhibition and tumor regression, accompanied by tolerable changes in body weight. These findings suggest that OP compounds possess potent efficacy and tolerable toxicity in ER+/HER2− breast cancer harboring overexpressed KAT6A, regardless of the status of ESR1 mutation. Notably, OP‐2 is the only KAT6 inhibitor to demonstrate dose‐dependent synergism with palbociclib and palazestrant (a complete ER antagonist) in T47D cells, indicating a new combination strategy for ER+ breast cancer. These findings further substantiate the oncogenic roles of KAT6A and KAT6B in ER+/HER2− breast cancer. However, comparative studies of different KAT6 inhibitors in human candidates are warranted to determine the optimal therapeutic strategy in the future.

Recently, OP‐3136 has been nominated as a development candidate to initiate phase I clinical trial in advanced or metastatic solid tumors (ClinicalTrials.gov Identifier: NCT06784193). Results are expected to expand the application of KAT6 inhibitors.

#### 5.4.5 Promising Pharmacy Combining KAT6A

Recently, several chemically induced proximity (CIP) technologies have undergone exploration, such as regulated induced proximity targeting chimeras, proteolysis targeting chimeras (PROTACs), and transcriptional or epigenetic chemical inducers of proximity [197, 213, 214, 215]. These heterobifunctional modalities function through the construction of a ternary complex, which is generally constituted by a ligand specific for the target protein enriched in tumors, a ligand binding an effector protein essential for cell viability, and a linker connecting these two ligands [216, 217]. The novel technologies enable direct on‐target effects toward cancer‐specific transcriptional reprogramming, which can offer an improved therapeutic window for inhibiting specific targets and reducing systemic adverse effects on host cells.

In the field of breast cancer, CIP approaches have led to the development of highly precise drugs that have demonstrated considerable antitumor efficacy and dose‐limiting toxicity. For example, the PROTAC ER degrader vepdegestrant (ARV‐471), a small molecule that eliminates disease‐causing ER proteins by recruiting ER through the potentiation of E3 ubiquitin ligase, has been shown to have favorable tolerability and robust ER degradation [218]. In the clinical trial known as VERITAC, ARV‐471 contributed to potent efficacy and manageable safety profiles in patients with locally advanced or metastatic ER+/HER2− breast cancer who had prior CDK4/6i therapy [219, 220]. Theoretically, the concept of CIP approaches for KAT6 complexes could work, especially for the KAT6A complex. The rationale is that KAT6A is overexpressed or amplified in ER+/HER2− breast cancer, which correlates with worse survival outcomes. This satisfies the most critical prerequisites for the formation of ternary complexes.

Indeed, PROTAC has been applied to the development of KAT6 inhibitors as a drug. A small‐molecule and ternary KAT6A:PROTAC:CRBN complex has been previously reported, wherein KAT6A was suggested to serve as a nonkinase target of compound iCDK9 (a selective CDK9 inhibitor) and its PROTAC bifunctional molecule CD‐5 [204]. The PROTAC‐stable isotope labeling using amino acids in cell culture experiment demonstrated that CD‐5 induced a robust and efficient degradation of KAT6A protein expression in a dose‐ and time‐dependent manner. This effect was also observed in the background of iCDK9 alone. The underlying molecular mechanism is assumed to be that the PROTAC bifunctional molecule of iCDK9 (as CD‐5) reduces the levels of H3K14Ac and H3K23Ac, thereby leading to the subsequent KAT6A degradation [204].

To date, the PROTAC technology utilized is still in the nascent stages. Additional explorations and improvements are necessary to facilitate the discovery and development of associated drugs in the real world.

### 5.5 Biomarkers for the Efficacy of KAT6 Inhibitors

It has been established that KAT6A directly modulates the transcriptional activity of ESR1 [32]. Consequently, numerous preclinical studies applied dynamic levels of ESR1 mRNA and monitored ERα protein expression as markers for KAT6A inhibition [180, 200, 201, 202, 203]. ESR1 mutations account for a common factor affecting ESR1 transcription in breast cancer [177], which predominantly occur in patients with HR+ breast cancer who have undergone a period of aromatase inhibitor (AI) treatment. The prevalence ranges from approximately 20 to 40%, varying depending on the location of metastases [177]. ESR1 mutations can affect the proliferation and metastasis of tumor cells by modulating the interaction between ERα protein and other signaling pathways, including the PI3K/AKT and MAPK signaling pathways. A wide variety of ESR1 mutations, such as D538G and Y537S, have been shown to correlate with drug resistance to ET (mostly AI) and CDK4/6i‐based therapy [221, 222, 223].

The transcriptional interaction between KAT6A and ESR1, along with the high frequency and the critical role of ESR1 mutations in ER+ breast cancer, has prompted researchers to investigate the potential impact of ESR1 mutations on the efficacy of KAT6 inhibitors. In the first‐in‐human clinical trial of the KAT6 inhibitor PF‐07248144, researchers performed preliminary biomarker analyses of circulating tumor DNA (ctDNA) mutation profiling at baseline, including ESR1 and PIK3CA/PTEN/AKT1 genes [37]. Plasma samples were collected from patients with ER+/HER2− breast cancer who were treated with PF‐07248144 in combination with fulvestrant and subsequently assessed. At baseline, 57% (24 out of 42) and 45.2% (19 out of 42) of the patients were detected to have at least one gene mutation in ESR1 and PIK3CA/PTEN/AKT1 genes, respectively.

For patients in the ESR1 mutant subgroup (*n* = 24) versus those in the ESR1 wild‐type (WT) subgroup (*n* = 18), the ORR (41.7 vs. 33.3%), CBR (50.0 vs. 55.5%), and the median PFS (10.7 vs. 10.9 months) were comparable [37, 224]. It indicates that ESR1 mutations at baseline have limited efficacy of the KAT6 inhibitor, despite the implication of KAT6A in ESR1 transcription. They further investigated dynamic changes in ctDNA and ESR1 mutations from baseline after 8 weeks of drug treatment. A substantial reduction in ctDNA and clearance of ESR1 mutants were observed in the majority of evaluable patients who received PF‐07248144 in combination with fulvestrant [37]. These findings indicate that changes in ctDNA and ESR1 mutational burden, rather than baseline ESR1 mutations, may serve as predictive markers for the efficacy of PF‐07248144 in combination with fulvestrant. Fulvestrant, one of the traditional SERDs, inhibits the growth of estrogen‐dependent tumors by inducing ER degradation via binding to ER [225]. Clinical studies have shown that the same dose of fulvestrant resulted in maintained PFS in patients with HR+/HER2− ABC irrespective of the status of ESR1 mutation [226, 227]. This indicates that fulvestrant remains an effective treatment option for breast cancer patients with ESR1 mutations alone. In another preclinical study, the KAT6 inhibitor OP‐2 displayed sustained antitumor activity in both wild‐type and ESR1‐mutated ER+ breast cancer cells [203]. It appears that we may need to consider whether KAT6 inhibition or fulvestrant is more effective in overcoming the negative effects of ESR1 mutations.

For patients in the PIK3CA/PTEN/AKT1 WT subgroup (*n* = 23), drug responses and survival outcomes were inclined to be superior to those who had PIK3CA/PTEN/AKT1 mutations (*n* = 19). Specifically, the ORR was 31.6 versus 43.5%, the CBR was 47.4 versus 56.5%, and the median PFS was 7.3 versus 13.7 months in the PIK3CA/PTEN/AKT1 mutant subgroup versus the PIK3CA/PTEN/AKT1 WT subgroup [37, 224]. Though the *p* values were not disclosed, the current trend indicates a correlation between the PIK3CA/PTEN/AKT1 mutations and the efficacy of the KAT6 inhibitor. In other types of tumors, KAT6A and KAT6B have been reported to mediate tumor proliferation and malignant progression through the PI3K/AKT signaling pathway, while inhibition of KAT6A and KAT6B results in decreased phosphorylation of PI3K and AKT [59, 64, 129, 228]. These findings suggest that dysregulation of the PI3K/AKT pathway may have the potential to influence the antitumor efficacy of KAT6 inhibitors. Therapeutic strategies for KAT6 inhibitors combined with small‐molecule compounds targeting PIK3CA/PTEN/AKT1 mutations may prove effective in overcoming KAT6 inhibitor resistance. Currently, PI3Kα inhibitors, including inavolisib and alpelisib, have demonstrated the capacity to induce durable disease control in patients with PIK3CA‐mutated breast cancer [229, 230, 231]. Meanwhile, antitumor treatment targeting AKT signaling pathways has yielded substantial advances in HR+/HER2− breast cancer. The first AKT inhibitor, capivasertib, has been approved for patients with HR+/HER2− ABC primarily on the basis of profound survival benefits presented in a phase III clinical trial called CAPItello‐291 [232]. Both PI3Kα inhibitors and AKT inhibitors improve drug responses and survival outcomes in the post‐CDK4/6i settings [229, 232, 233]. Emerging evidence in the phase I clinical trial supports KAT6 inhibitor PF‐07248144 as a promising option for patients with ER+/HER2− ABC who have progressed after receiving CDK4/6i therapy. One reality to consider is that the development of KAT6 inhibitors lags far behind PI3Kα inhibitors and AKT inhibitors at present. It is anticipated that the forthcoming clinical data concerning KAT6 inhibitors in the field of breast cancer will facilitate the application of KAT6 inhibitors and reform the current treatment patterns of breast cancer.

Based on current data, changes in ctDNA and ESR1 variant allele frequency, in addition to PIK3CA/PTEN/AKT1 mutations at baseline, may be used to predict the efficacy of PF‐07248144 in combination with fulvestrant. Nevertheless, the viewpoints are derived from a preliminary analysis of the only in‐human clinical study, which has limited samples for ctDNA testing. Further explorations are necessary to elucidate the mechanism of resistance to KAT6 inhibitors and to develop biomarkers for predicting the efficacy of KAT6 inhibitors in clinical settings.

## 6 Challenges and Future Directions

KAT6A and KAT6B represent attractive epigenetic targets in the context of breast cancer biology. In light of the encouraging outcomes observed with KAT6 inhibitors in ER+ breast cancer, it can be inferred that these small‐molecule inhibitors have the potential to deliver new therapeutic strategies for the treatment of ER+/HER2− breast cancer. Nevertheless, it is imperative to consider several factors when developing therapeutic strategies.

### 6.1 Unresolved Questions

Given the crucial role of KAT6 in physiological processes involving hematopoiesis, tissue development, and immunological regulation, some side effects accompanied by antitumor activity from KAT6 inhibitors could act as negative factors for drug development. Although the safety profile of KAT6 inhibitor PF‐07248144 in the phase 1 clinical trial has been deemed tolerable and manageable, a more comprehensive understanding is required to achieve an optimal balance between the antitumor efficacy and safety profiles of different on‐target inhibition modalities. This will promote the identification of the most practical and safe treatment approach.

Besides, resistance to KAT6 inhibitors and postresistance treatment strategies are factors to consider. Some individuals diagnosed with breast cancer develop drug resistance to antitumor treatment after a certain period of effectiveness, while others display poor drug response at the outset [234]. This emphasizes the necessity of exploring mechanisms of resistance to corresponding anticancer drugs in an effort to identify drug‐sensitive individuals and avoid unnecessary toxicity for patients who are not responsive. Regarding KAT6 inhibitors, research on therapeutic approaches following resistance to these inhibitors may be more readily pursued, focusing on the genes or pathways implicated in drug resistance. Sequencing technologies based on ctDNA profiling, next‐generation sequencing, and digital spatial profiling may provide possible directions.

### 6.2 Technological Innovations

Developing more precise KAT6 inhibitors through emerging CIP approaches may contribute to alleviating AEs and improving antitumor efficacy. Admittedly, the currently reported KAT6 inhibitors have displayed prominent advances in selectivity, bioavailability, and clearance compared with a series of epigenetic drugs in the past. Application of CIP approaches to inhibit or degrade KAT6A is thought to achieve better tumor‐specific targeting of KAT6A and broaden the therapeutic window for KAT6A suppression.

As we previously documented, a PROTAC‐based drug targeting KAT6A has been developed to show profound degradation of KAT6A in preclinical models. Another novel technology, labeled as acetylation‐targeting chimeras (AceTACs), is also a promising approach. It has been proposed that other members of the KAT family recruit HAT p300/CBP to acetylate the p53 tumor suppressor protein [235]. AceTACs exert pharmacological effects via a precise and direct impact on the acetylation of a target protein, which is responsible for a chemically directed interaction with an acetyltransferase. As pivotal members of the KAT family, KAT6 proteins play essential roles in mediating acetylation, which has been reported to promote the development of cancers. Theoretically, KAT6 could serve as a potential target protein for the development of AceTAC‐based therapeutics.

### 6.3 Translational Opportunities

Based on current preclinical and clinical evidence, the primary target population for KAT6 inhibitors is expected to be patients with ER+/HER2− breast cancer. ER+/HER2− breast cancer represents the most prevalent subtype, accounting for approximately 60% of all cases [236]. ET is the therapeutic foundation for this particular subtype of breast cancer, which generally contributes to lower recurrent risks and improved survival outcomes [211, 237]. Since the approval of CDK4/6i, the combination of CDK4/6i and ET has developed as a new standard‐of‐care treatment regimen, replacing the traditional treatment modality of single‐agent ET for many patients [238, 239, 240]. As the number of patients receiving CDK4/6i plus ET increases, the emergence of CDK4/6i resistance has become an undeniable issue [182]. Resistance to CDK4/6i develops in a subset of early‐stage and nearly all advanced‐stage patients, ultimately leading to disease progression. This underscores the importance of exploring new druggable targets and developing associated treatment strategies to overcome drug resistance to CDK4/6i‐based therapy. According to data emerging from the only phase I clinical trial, KAT6 inhibitor plus fulvestrant represents a promising and effective therapeutic strategy for ER+/HER2− patients who have developed resistance to CDK4/6i and ET.

Furthermore, an emerging translational study has provided additional insights for selecting patients who can benefit from KAT6 inhibitors based on biomarkers. The ctDNA profiling from peripheral blood testing in the first‐in‐human phase I clinical trial of the KAT6 inhibitor showed that patients with wild‐type PIK3CA/PTEN/AKT1 derived improved ORR, CBR, and PFS [37]. Although it is derived from the preliminary biomarker analyses, it provides a direction for researchers to develop practical therapeutic strategies focused on targets associated with drug resistance. It suggests that patients harboring mutations in PIK3CA, PTEN, or AKT1 may be resistant to KAT6 inhibitor‐based therapeutic strategies.

Given the specific inhibitory effect of KAT6 on ER, an ideal development strategy should involve ET combination, rather than monotherapy of KAT6 inhibitors. The current phase I clinical trial supports this combination strategy, which exhibits a favorable safety profile and promising antitumor efficacy. Furthermore, emerging evidence has demonstrated synergistic efficacy between KAT6 inhibitors and Menin inhibitors in preclinical models of ET‐resistant ER+ breast cancer, with a therapeutic effect comparable to that of the novel SERD elacestrant [132]. These results may provide a rationale for future clinical development of such combination strategies in humans.

Overall, KAT6A and KAT6B are attractive and desirable epigenetic targets for therapeutic leverage in human disease, particularly in ER+/HER2− breast cancer. It is anticipated that KAT6 inhibitors will extend therapeutic benefits to a broader range of cancer patients in the future, with further improvements in the management of AEs, the development of novel agents through emerging technologies, and the therapeutic strategies to overcome drug resistance.

## Author Contributions

Conceptualization: FM, JW, and YT. Writing—original draft: YT. Writing—review and editing: FM, JW, and YT. Funding acquisition and supervision: FM. All authors read, critically revised, and approved the final manuscript.

## Funding

The study was supported by the CAMS Innovation Fund for Medical Sciences (2022‐I2M‐2‐001), the National Natural Science Foundation of China (82172875), and the National Natural Science Foundation of China (82230058).

## Ethics Statement

The authors have nothing to report.

## Conflicts of Interest

The authors declare no conflicts of interest.

## Data Availability

Data are available from the corresponding author with reasonable request.
